# Digital Health Engagement Behaviors in US Family Caregivers: Trend Analysis Using the Health Information National Trends Survey 2019-2022 Data

**DOI:** 10.2196/94390

**Published:** 2026-09-03

**Authors:** Lena Lee, Elisa Son, Li Yang, Chantal Gerrard, Jenny Yarmovsky, Gwenyth Wallen

**Affiliations:** 1 Translational Biobehavioral and Health Promotion, National Institutes of Health (NIH) Clinical Center, 10 Center Drive, Bethesda, MD, 20892-1151, United States, 1 301-451-1266

**Keywords:** caregivers, digital health engagement, health information seeking, social media, online medical records

## Abstract

**Background:**

Digital health has provided caregivers with access to supportive resources without space-time restrictions. Caregivers’ digital health engagement behaviors can help them track their own health and that of care recipients as well as communicate with others. While digital health tools have become more prevalent since the COVID-19 pandemic, the trend in caregiver engagement has been less explored.

**Objective:**

This study examined the trends and factors associated with selected digital health engagement behaviors in family caregivers in the United States, using the HINTS (Health Information National Trends Survey) datasets collected from HINTS 5 Cycles 3 and 4 and HINTS 6 from 2019 to 2022.

**Methods:**

Our cross-sectional data analysis included 1676 family caregivers. Dependent variables were (1) access to online medical records (caregivers’ and care recipients’) and (2) health-related use of social media (sharing health information, interacting with others, and watching health-related videos). Independent variables were survey cycles, demographic, socioeconomic, caregiving, and internet technology factors. Weighted multivariable logistic regression analyses were conducted.

**Results:**

Among 1676 caregivers (HINTS 5 Cycle 3: n=570; HINTS 5 Cycle 4: n=412; HINTS 6: n=694), access to online medical records increased from HINTS 5 Cycle 3 in 2019 to HINTS 6 in 2022. Access to caregivers’ own records rose from 48.7% to 72.6% (*P*<.001), and access to care recipients’ records increased from 30.8% to 44.5% (*P*<.001). Health-related social media use also increased, including sharing health information (22.5% vs 39.1%; *P*<.001), interacting with others (16.6% vs 27.0%; *P*<.001), and watching health-related videos (49.5% vs 60.9%; *P*=.005). In adjusted analyses, higher education (≥ college graduate vs ≤ high school: odds ratio [OR] 2.75, 95% CI 1.56‐4.85; *P*<.001) and having health insurance (OR 2.40, 95% CI 1.24‐4.68; *P*=.010) were associated with access to caregivers’ records. Female participants (OR 1.96, 95% CI 1.36‐2.84; *P*<.001) and spousal caregiving (OR 2.14, 95% CI 1.26‐3.65; *P*=.005) were associated with access to care recipients’ records. High-speed internet access was strongly associated with digital health engagement outcomes (eg, sharing health information: OR 3.98, 95% CI 2.15‐7.35; *P*<.001).

**Conclusions:**

Digital health engagement—including access to online medical records and the use of social media for health-related purposes—among US family caregivers increased following the COVID-19 pandemic. These findings suggest that health care professionals and researchers should consider multifaceted factors, such as age, race/ethnicity, geography, education, insurance coverage, and digital access, when designing and implementing digital health tools and technology-based interventions. Future research should evaluate how digital technologies and the automation of systems “talking” to other systems, including AI, can better support caregivers’ health information needs and care coordination. The varying learning curves for individuals and groups could be further explored for effective and efficient adoption and usage.

## Introduction

As life expectancy has increased and chronic diseases have become more prevalent, unpaid caregiving has risen in the United States (US). It is estimated that approximately 53.0 million adults provided unpaid care for an adult or a child with special needs [1]. Caregiving tasks include, but are not limited to, assistance with daily living, care coordination, emotional support, and gathering treatment-related information [2,3]. While caregiving can be a rewarding experience, family caregivers are often unprepared for the changes in their roles and responsibilities, leading to physical, emotional, social, and financial burdens [4,5]. The burden of caregiving may also disrupt caregivers’ self-care, with one in four caregivers finding it difficult to take care of their own health [1,6]. Prolonged exposure to the burden associated with caregiving could lead to increased morbidity and mortality among caregivers, which may, in turn, impact the health and well-being of the care recipients [5,6].

Caregivers often require supportive information and resources for caring for their care recipients and for their own health. For example, access to medical records can allow them to monitor their health status and receive clinical advice from health care providers [7]. Online resources for care coordination can support caregivers as they navigate the health care system, and resources for medical or nursing tasks can help them practice caregiving activities [7,8]. Caregivers also appreciate the opportunity to share experiences with peer caregivers to reduce the burden of caregiving [8,9]. The proliferation of digital health has provided opportunities to access supportive information and resources and to communicate with health care providers and peer caregivers without space-time restrictions [10,11]. Digital health is a broad concept that refers to the use of information and communication technologies, such as mobile health and telehealth, to facilitate health care delivery and promote wellness [12,13]. Digital health has grown over the past decades, and its importance has further increased since the COVID-19 pandemic, as most physician visits shifted to telemedicine encounters and a substantial portion of health services transitioned to web-based platforms [11,14].

Family caregivers’ engagement with digital health (hereafter, digital health engagement behaviors) can support managing the health of their care recipients and themselves while facilitating the coordination of care services [4,8,10]. Digital health engagement behaviors refer to the extent and patterns of individuals’ engagement with digital technologies and online platforms for health-related information seeking, communication, social support, health management, access to medical records, and health-related social media use [15]. One digital health tool readily available for caregivers is access to online medical records through patient portals. As more health care systems and providers adopt electronic health records and tools for managing electronic protected health information, patients and their families can easily access medical records through digital platforms [16]. Granting caregivers access to patient portals allows them to review medical records and promotes active communication with health care providers [7]. According to the HINTS (Health Information National Trends Survey), which is a nationally representative survey conducted by the National Cancer Institute, 57% of US adults accessed their online medical records in HINTS 6 (2022), compared to 37% in HINTS 5 Cycle 3 (2019) and 38% in HINTS 5 Cycle 4 (2020) [17]. Individuals’ access to online medical records can be influenced by various demographic and contextual factors. The literature indicates that older age, rural residence, and racial and ethnic minority status are often associated with lower access to online medical records, whereas higher socioeconomic status (eg, higher education, income, and health insurance coverage) and better access to internet technology (eg, high-speed internet and smart devices) are considered facilitators [18,19]. For family caregivers, caregiving-related characteristics, such as the caregiver relationship and the care recipient’s condition, may influence the level of engagement [7,20]. However, the extent to which caregivers access online medical records for themselves and their care recipients, as well as the associated factors, has not been fully explored.

Another prominent digital health engagement behavior is using social media for health-related purposes. Social media refers to digital communication platforms where people share information, content, or ideas and interact with others via websites and mobile apps [21]. Over the past decade, social media platforms have evolved rapidly, leading to a dramatic increase in the number of global users from 970 million in 2010 to 5.24 billion by 2025 [22]. The use of social media for health-related purposes has further expanded, especially since the outbreak of the COVID-19 pandemic [23]. However, this trend remains less explored among family caregivers. Existing studies on caregivers’ use of social media have primarily focused on their participation in online support groups related to the care recipient’s medical condition [9,24]. Through social media, caregivers receive informational and emotional support, as well as a sense of social belonging, by exchanging advice and information about the disease and sharing caregiving experiences with others in similar situations [9,24]. As social media applications become more diverse, caregivers can now engage with them in both caregiving and personal contexts [25,26]. Although research in this area is still emerging, multiple characteristics could be associated with health-related use of social media among caregivers. In the literature, younger and female caregivers are generally more likely to use social media for health information and support [23]. Some studies also indicate that racial and ethnic minorities are active users of social media [23]. Higher socioeconomic status and greater access to internet technology tend to facilitate this behavior [22,23]. While it is assumed that caregiving-related factors may also impact social media use, these factors have been less studied among caregivers compared to the general population.

Digital tools can be useful for family caregivers by supporting the caregiving role and caring for their own health. Understanding caregivers’ digital health engagement behaviors before, during, and after the pandemic and the associated characteristics may help guide future strategies for leveraging digital tools to support caregivers’ needs. This study aimed to examine the prevalence of, and factors associated with, selected digital health engagement behaviors—access to online medical records and health-related use of social media—in US family caregivers, using national-level datasets collected from HINTS 5 Cycle 3 (2019), HINTS 5 Cycle 4 (2020), and HINTS 6 (2022).

## Methods

### Study Design, Data Source, and Study Population

We conducted a cross-sectional data analysis merging 3 cycles of HINTS: HINTS 5 Cycle 3 collected between January 2019 and May 2019 (n=5438), HINTS 5 Cycle 4 collected between February 2020 and June 2020 (n=3865), and HINTS 6 collected between March 2022 and November 2022 (n=6252), noting that data were not collected in 2021. We targeted respondents who identified themselves as nonprofessional, unpaid caregivers. HINTS is a nationally representative survey that has been administered by the National Cancer Institute since 2003. The survey targets noninstitutionalized adults in the United States and collects data on their need for, and access to, health-related information and health-related behaviors. The survey used a 2-stage stratified random sampling method. The sampling frame was derived from a database of addresses maintained by Marketing Systems Group. All nonvacant US residential addresses in the Marketing Systems Group database were eligible for sampling. In the first stage, a stratified random sample of residential addresses was drawn from the database. In the second stage, 1 adult was selected from each sampled household using the Next Birthday Method. Details on the sampling method and data collection are available in the methodology reports for each HINTS cycle [27-29]. For this study, we focused on respondents who identified themselves as nonprofessional, unpaid caregivers. This study was reported in accordance with the STROBE (Strengthening the Reporting of Observational Studies in Epidemiology) guidelines (Checklist 1).

### Variables of Interest

#### Dependent Variables: Digital Health Engagement Behaviors

We included two types of dependent variables in our analysis: (1) access to online medical records and (2) health-related use of social media. The variables for access to online medical records were derived from 2 questions: “How many times did you access your online medical record or patient portal in the last 12 months?” and “How many times did you access that person’s (care recipient’s) online medical record in the last 12 months?” The distributions of both variables were highly skewed, with relatively few respondents in the higher-frequency categories. Therefore, responses were dichotomized to indicate any access (≥1 time, coded as “yes”) vs no access (0 times, coded as “no”). The variables of health-related social media use were the following behaviors: sharing personal or general health information, interacting with others in an online forum or support group for people with similar health or medical issues, and watching health-related videos. If a respondent engaged in each of the behaviors in the past 12 months, it was coded as “yes”; if not, it was coded as “no.”

#### Independent Variables

##### Demographic and Health-Related Factors

Demographic and health-related factors included age group (18‐34, 35-49, 50‐64, and 65 y or older), sex (male and female), race/ethnicity (non–Hispanic White, non–Hispanic Black, Hispanic, non–Hispanic Asian, or other), marital status (married or living with a partner, and not married), confidence in the ability to take care of their health (completely or very confident, and less or not confident), cancer history (yes or no), and having any of the following medical conditions (yes or no): diabetes, hypertension, heart condition, and/or lung disease. Psychological distress was assessed using the Patient Health Questionnaire-4, with scores ranging from 0 to 12; higher scores indicate greater levels of anxiety and depression.

##### Socioeconomic Factors

Socioeconomic factors included being covered by health insurance (yes or no), education level (≤high school graduate, some college, and ≥college graduate), annual household income (US <$35,000, $35,000-$74,999, and ≥$75,000), and residential areas (rural and urban). Residential area was based on the 2013 Rural-Urban Continuum Codes, ranging from 1 to 9, as defined by the US Department of Agriculture. We recategorized codes 1 to 3 as urban areas and codes 4 to 9 as rural areas.

##### Caregiving Factors

Caregiving factors included variables regarding relationships with care recipients and their caregiving conditions. Variables of caregiving relationships were as follows: caring for child/children (yes or no), caring for parent(s) (yes or no), and/or caring for spouse/partner (yes or no). The variables of caregiving conditions of care recipients’ health status were: cancer (yes or no); Alzheimer’s disease, neurological/developmental, or mental health issues (yes or no); orthopedic or musculoskeletal, or aging issues (yes or no); acute conditions (yes or no); and/or chronic conditions (yes or no).

##### Internet Technology Factors

The variables of whether having access to a high-speed internet service (yes or no), having a tablet computer (yes or no), and having a smartphone (yes or no) were included.

### Statistical Analysis

Descriptive statistics (means, SDs, frequencies, and percentages) were used to describe the respondents’ characteristics and digital health engagement behaviors in each HINTS cycle. Differences among the survey cycles were examined using Rao-Scott adjusted chi-square tests. Controlling for survey cycles, age, and sex, unadjusted logistic regression tests were conducted to examine the associations between digital health engagement behaviors and each independent variable. Independent variables with a *P* value<.10 in the unadjusted regression analyses were retained in the multivariable logistic regression models. The final models were selected using backward elimination with a removal criterion of *P* value<.10. We merged the datasets using IBM SPSS Statistics version 30 and performed all analyses using R version 4.6.0 (R Foundation for Statistical Computing). We applied the final sample weight and replicate weights to the analyses to obtain nationally representative estimates and SEs.

### Ethical Considerations

The Westat Institutional Review Board (IRB) approved HINTS 6 (project number 6632.03.51) and HINTS 5 Cycle 3 and HINTS 5 Cycle 4 (project number 6048.14). A “Not Human Subjects Research” determination was given to HINTS 5 from the National Institutes of Health Office of Human Subjects Research (Exempt number 13204) and HINTS 6 from the National Institutes of Health Office of IRB Operations (IRB ID: IRB002042).

## Results

### Descriptive Characteristics

**Figure 1.**
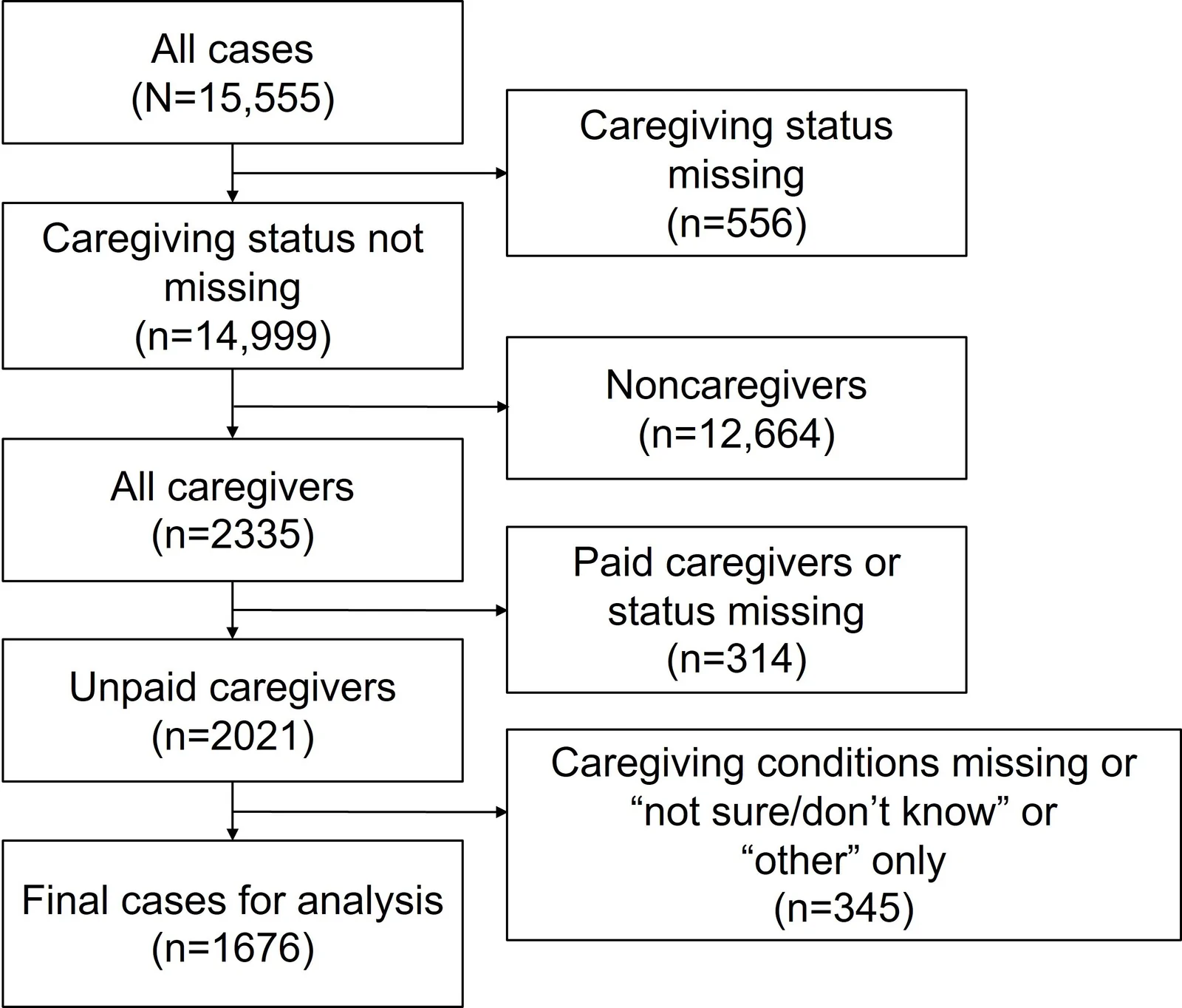
Sample selection flow.

Of the total respondents from the 3 cycles of HINTS (N=15,555), we selected those who fulfilled the following criteria: (1) answered “yes” to the question “are you currently caring for or making health care decisions for someone with a medical, behavioral, disability, or other condition?”; (2) answered “no” to the question “do you provide any of this care professionally as part of a job (eg, as a nurse or professional home health aide)?”; and (3) did not respond to the caregiving condition as “not sure/don’t know” only or “other” only. After excluding cases that did not meet the criteria, we included 1676 caregivers in the analysis (weighted N=91,486,345.9): 570 from HINTS 5 Cycle 3, 412 from HINTS 5 Cycle 4, and 694 from HINTS 6. The flow of the sample selection is shown in Figure 1.

Descriptive characteristics of the pooled sample (N=1676) are presented in Table 1. Approximately two-thirds of the sample were female participants (n=109, 59.8%), aged 50 years or older (n=1169, 61.5%), and non–Hispanic White (n=956, 64.9%). The majority were married (n=1083, 69.8%), had some college or higher level of education (n=1287, 76.8%), lived in urban areas (n=1483, 87.1%), and were covered by health insurance (n=1565, 92.8%). A similar percentage of the sample reported that they were caregiving for a child (n=523, 38.7%) and/or a parent (n=630, 37.8%). The most reported condition for caregiving was Alzheimer disease and other neurological and mental health issues (n=1055, 61.7%), followed by orthopedic and aging-related issues (n=709, 41.8%). Most of the sample had access to high-speed internet (n=1379, 84.4%) and owned a smartphone (n=1471, 91.4%) and/or a tablet (n=1066, 68.1%). The descriptive characteristics did not differ significantly between the survey cycles.

**Table 1.** Descriptive characteristics of the pooled sample (unweighted N=1676)^a^.

Characteristics	Total (N=1676), n (%)	HINTS^b^ 5 Cycle 3 (2019; n=570), n (%)	HINTS 5 Cycle 4 (2020; n=412), n (%)	HINTS 6 (2022; n=694), n (%)	*P* value
Sex					.47
Male	545 (40.2)	204 (39.4)	125 (37.4)	216 (43.5)	
Female	1095 (59.8)	359 (60.6)	280 (62.6)	456 (56.5)	
Age group (y)					.22
18‐34	124 (11.1)	38 (10.3)	27 (9.0)	59 (13.9)	
35‐49	354 (27.4)	117 (28.9)	103 (31.2)	134 (22.5)	
50‐64	667 (43.2)	243 (44.9)	160 (43.7)	264 (41.3)	
≥65	502 (18.3)	163 (15.9)	111 (16.1)	228 (22.3)	
Race/ethnicity					.81
Non–Hispanic White	956 (64.9)	336 (63.1)	253 (68.5)	367 (63.1)	
Non–Hispanic Black	213 (8.8)	67 (7.9)	41 (8.8)	105 (9.5)	
Hispanic	237 (13.7)	73 (15.8)	55 (10.6)	109 (14.7)	
Non–Hispanic Asian or other	146 (12.6)	48 (13.2)	31 (12.1)	67 (12.6)	
Marital status					.64
Unmarried^c^	546 (30.2)	177 (27.4)	132 (31.2)	237 (31.7)	
Married	1083 (69.8)	381 (72.6)	271 (68.8)	431 (68.3)	
PHQ-4^d^ score					.39
Normal (0‐2)	1045 (61.8)	382 (66.7)	254 (57.6)	409 (61.5)	
Mild (3-5)	355 (20.7)	100 (15.2)	87 (22.9)	168 (23.2)	
Moderate (6-8)	149 (9.9)	41 (10.0)	41 (11.1)	67 (8.8)	
Severe (9-12)	91 (7.6)	35 (8.0)	23 (8.4)	33 (6.5)	
Ability to take care of health					.63
Completely or very confident	1174 (65.7)	406 (68.5)	291 (65.2)	477 (63.6)	
Less or not confident	498 (34.3)	164 (31.5)	121 (34.8)	213 (36.4)	
Cancer history					.63
No	1361 (87.8)	471 (89.3)	323 (86.8)	567 (87.3)	
Yes	285 (12.2)	93 (10.7)	83 (13.2)	109 (12.7)	
Medical conditions^e^					.14
No	671 (43.0)	246 (47.3)	151 (37.6)	274 (44.5)	
Yes	990 (57.0)	320 (52.7)	257 (62.4)	413 (55.5)	
Education					.78
≤High school graduate	302 (23.2)	90 (21.9)	84 (24.0)	128 (23.8)	
Some college	508 (44.8)	168 (44.7)	131 (47.0)	209 (42.6)	
≥College graduate	831 (32.0)	306 (33.4)	188 (29.0)	337 (33.7)	
Residential area					.74
Rural	193 (12.9)	59 (11.7)	47 (14.0)	87 (13.0)	
Urban	1483 (87.1)	511 (88.3)	365 (86.0)	607 (87.0)	
Health insurance					.16
No	100 (7.2)	30 (8.0)	17 (4.9)	53 (8.8)	
Yes	1565 (92.8)	533 (92.0)	393 (95.1)	639 (91.2)	
Annual income (US $)					.26
<35,000	372 (24.1)	111 (20.9)	109 (29.6)	152 (21.6)	
35,000-74,999	489 (29.6)	171 (28.6)	111 (27.8)	207 (32.4)	
≥75,000	659 (46.3)	238 (50.6)	157 (42.7)	264 (46.1)	
Caregiving: child					.11
No	1153 (61.3)	394 (60.8)	254 (56.8)	505 (66.2)	
Yes	523 (38.7)	176 (39.2)	158 (43.2)	189 (33.8)	
Caregiving: spouse					.73
No	1235 (77.3)	424 (79.3)	309 (76.8)	502 (75.9)	
Yes	441 (22.7)	146 (20.7)	103 (23.2)	192 (24.1)	
Caregiving: parent					.10
No	1046 (62.2)	346 (55.5)	267 (65.0)	433 (65.4)	
Yes	630 (37.8)	224 (44.5)	145 (35.0)	261 (34.6)	
Caregiving: cancer					.89
No	1486 (89.7)	506 (88.9)	363 (90.0)	617 (90.1)	
Yes	190 (10.3)	64 (11.1)	49 (10.0)	77 (9.9)	
Caregiving: Alzheimer’s disease and others^f^					.51
No	621 (38.3)	195 (34.7)	147 (39.8)	279 (40.1)	
Yes	1055 (61.7)	375 (65.3)	265 (60.2)	415 (59.9)	
Caregiving: orthopedic and others^g^					.69
No	967 (58.2)	308 (57.2)	215 (56.8)	444 (60.5)	
Yes	709 (41.8)	262 (42.8)	197 (43.2)	250 (39.5)	
Caregiving: chronic conditions^h^					.94
No	1001 (60.9)	337 (59.9)	243 (61.2)	421 (61.4)	
Yes	675 (39.1)	233 (40.1)	169 (38.8)	273 (38.6)	
Caregiving: acute conditions^i^					.12
No	1478 (88.4)	526 (91.7)	375 (88.6)	577 (85.2)	
Yes	198 (11.6)	44 (8.3)	37 (11.4)	117 (14.8)	
Access to high-speed internet					.33
No	250 (15.6)	70 (13.3)	63 (14.7)	117 (18.4)	
Yes	1379 (84.4)	487 (86.7)	327 (85.3)	565 (81.6)	
Having a tablet computer					.49
No	594 (31.9)	197 (30.5)	153 (34.9)	244 (30.2)	
Yes	1066 (68.1)	364 (69.5)	255 (65.1)	447 (69.8)	
Having a smartphone					.46
No	189 (8.6)	79 (10.6)	46 (8.2)	64 (7.2)	
Yes	1471 (91.4)	482 (89.4)	362 (91.8)	627 (92.8)	

a The final sample weight and replicate weights are applied.

b HINTS: Health Information National Trends Survey.

c Unmarried refers to divorced, widowed, separated, or never married.

d PHQ-4: Patient Health Questionnaire-4.

e Medical conditions such as diabetes, hypertension, heart condition, and/or lung disease.

f Caregiving: Alzheimer’s disease and other conditions such as confusion, dementia, forgetfulness, neurological/developmental issues, and/or mental health/behavioral/substance abuse issues.

g Caregiving: orthopedic and other conditions such as orthopedic/musculoskeletal issues, and/or aging/aging–related health issues.

h Caregiving: chronic conditions such as hypertension, diabetes, heart disease, and/or lung disease.

i Caregiving: acute conditions such as recovery from surgery or an injury.

### Digital Health Engagement Behaviors

Table 2 presents the selected digital health engagement behaviors for each survey cycle, with significant differences found between the cycles. The percentage of caregivers accessing their own online medical records increased from around 50% (278/570) in HINTS 5 Cycle 3 (2019) and HINTS 5 Cycle 4 (2020) to 72.6% (487/694) in HINTS 6 (2022*; P*<.001). Compared to HINTS 5 Cycle 3 (2019; 168/570, 30.8%), the percentage of caregivers accessing the care recipient’s online medical records decreased in HINTS 5 Cycle 4 (2020; 106/412, 22.7%) but increased in HINTS 6 (2022; 307/694, 44.5%; *P*<.001). Regarding health-related use of social media, the percentages of caregivers sharing health information decreased in HINTS 5 Cycle 4 (2020; 74/412, 17.9%), then increased in HINTS 6 (2022; 263/694, 39.1%), compared to HINTS 5 Cycle 3 (2019; 110/570, 22.5%; *P*<.001). Interacting with others online (69/570, 16.6% in 2019; 55/412, 12.2% in 2020; and 176/694, 27.0% in 2022; *P*<.001) and watching health-related videos (238/570, 49.5% in 2019; 184/412, 45.6% in 2020; and 419/694, 60.9% in 2022; *P*=.005) showed similar trends.

**Table 2.** Digital health engagement behaviors of the pooled sample (unweighted N=1676)^a^.

Behaviors	Total (N=1676), n (%)	HINTS^b^ 5 Cycle 3 (2019; n=570), n (%)	HINTS 5 Cycle 4 (2020; n=412), n (%)	HINTS 6 (2022; n=694), n (%)	*P* value
Access to online medical records					
Caregiver’s records					<.001
No	682 (42.5)	282 (51.3)	206 (50.1)	194 (27.4)	
Yes	969 (57.5)	278 (48.7)	204 (49.9)	487 (72.6)	
Care recipient’s records					<.001
No	1069 (67.0)	399 (69.2)	290 (77.3)	380 (55.5)	
Yes	581 (33.0)	168 (30.8)	106 (22.7)	307 (44.5)	
Health-related social media use					
Sharing health information					<.001
No	1200 (73.2)	453 (77.5)	331 (82.1)	416 (60.9)	
Yes	447 (26.8)	110 (22.5)	74 (17.9)	263 (39.1)	
Interacting with people^c^					<.001
No	1358 (81.2)	496 (83.4)	351 (87.8)	511 (73.0)	
Yes	300 (18.8)	69 (16.6)	55 (12.2)	176 (27.0)	
Watching health-related videos					.005
No	818 (47.8)	327 (50.5)	222 (54.4)	269 (39.1)	
Yes	841 (52.2)	238 (49.5)	184 (45.6)	419 (60.9)	

a Final sample weight and replicate weights are applied.

b HINTS: Health Information National Trends Survey.

c Interacting with others in online forums or support groups for people with similar health or medical issues.

### Associations of Survey Cycle and Covariates With Digital Health Engagement Behaviors

#### Access to Online Medical Records

**Figure 2.**
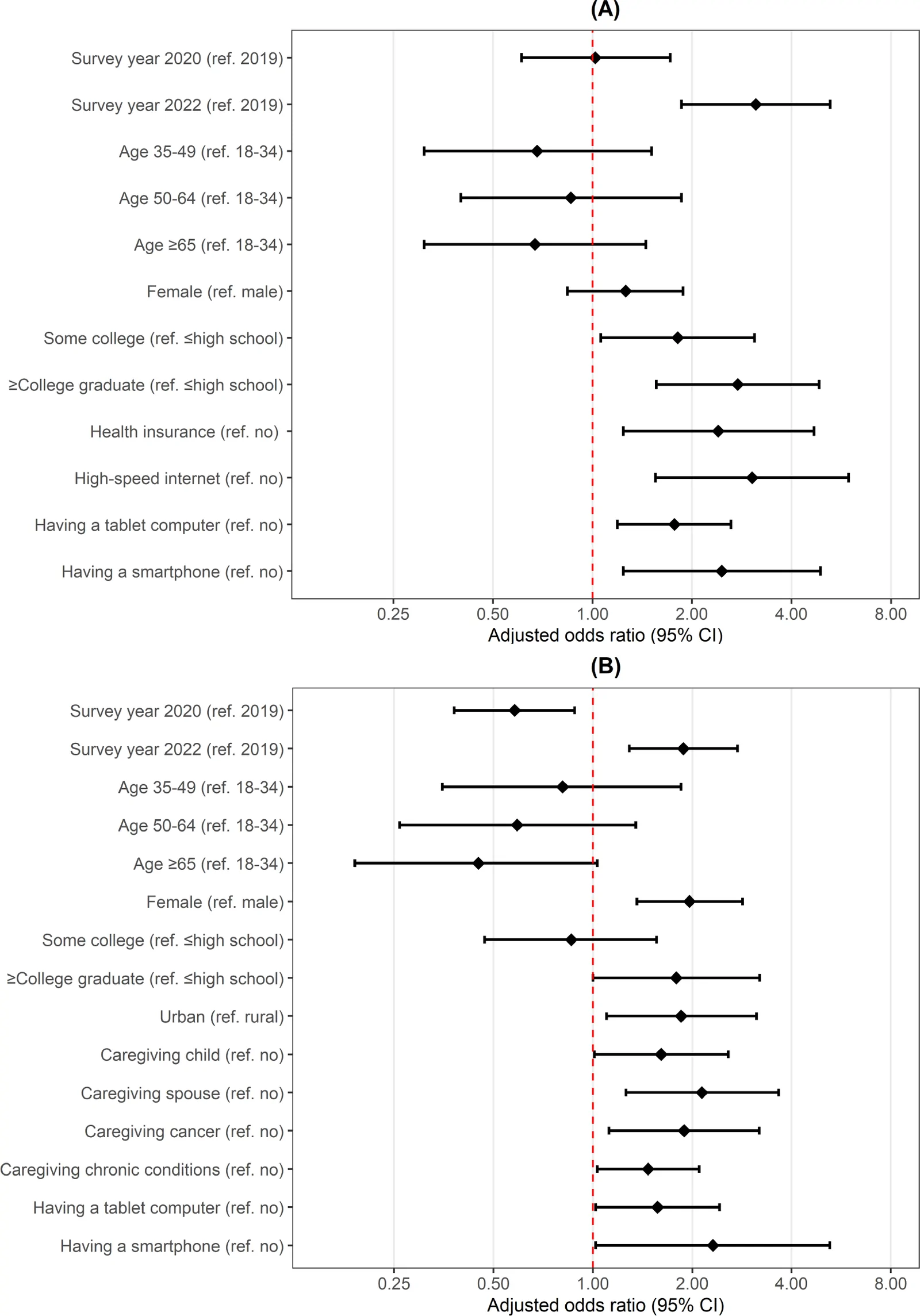
Forest plots of the association between survey cycle/covariates and access to online medical records. (A) Caregiver’s online medical records and (B) care recipient’s online medical records.

Compared with HINTS 5 Cycle 3 (2019), caregivers in HINTS 6 (2022) were more likely to access their own online medical records (adjusted odds ratio [aOR] 3.12, 95% CI 1.86‐5.24; *P*<.001) and those of care recipients (aOR 1.88, 95% CI 1.29‐2.74; *P*=.001). Caregivers with some college education (aOR 1.81, 95% CI 1.06‐3.09; *P*=.03) or higher education (aOR 2.75, 95% CI 1.56‐4.85; *P*<.001), health insurance coverage (aOR 2.40, 95% CI 1.24‐4.68; *P*=.01), access to high-speed internet (aOR 3.04, 95% CI 1.55‐5.95; *P*=.001), and having a tablet computer (aOR 1.77, 95% CI 1.19‐2.62; *P*=.005) and/or a smartphone (aOR 2.46, 95% CI 1.24‐4.89; *P*=.01) were more likely to access their own online medical records compared with their counterparts. Being a female caregiver (aOR 1.96, 95% CI 1.36‐2.84; *P*<.001), living in an urban area (aOR 1.85, 95% CI 1.10‐3.13; *P*=.02), caring for a child (aOR 1.61, 95% CI 1.01‐2.57; *P*=.046) or spouse (aOR 2.14, 95% CI 1.26‐3.65; *P*=.005), caring for individuals with cancer (aOR 1.89, 95% CI 1.12‐3.19; *P*=.02) or chronic conditions (aOR 1.47, 95% CI 1.03‐2.10; *P*=.04), and having a tablet computer (aOR 1.57, 95% CI 1.02‐2.42; *P*=.04) and/or a smartphone (aOR 2.31, 95% CI 1.02‐5.22; *P*=.04) were associated with a higher likelihood of accessing the care recipient’s online medical records compared with their counterparts. The aORs with 95% CIs and Nagelkerke pseudo-*R*^2^ of logistic regression models are shown in Table 3, with forest plots displayed in Figure 2.

**Table 3.** Multivariable logistic regression of access to online medical records^a^.

Variables	Caregiver’s records^b^, OR^c^ (95% CI)	*P* value	Care recipient’s records^d^, OR (95% CI)	*P* value
Survey cycles				
HINTS^e^ 5 Cycle 3 (2019; reference)	—^f^	—	—	—
HINTS 5 Cycle 4 (2020)	1.02 (0.61‐1.72)	.93	0.58 (0.38‐0.88)	.01
HINTS 6 (2022)	3.12 (1.86‐5.24)	<.001	1.88 (1.29‐2.74)	.001
Age group (y)				
18‐34 (reference)	—	—	—	—
35‐49	0.68 (0.31‐1.51)	.34	0.81 (0.35‐1.85)	.61
50‐64	0.86 (0.40‐1.86)	.71	0.59 (0.26‐1.35)	.21
≥65	0.67 (0.31‐1.45)	.31	0.45 (0.19‐1.03)	.06
Sex				
Male (reference)	—	—	—	—
Female	1.26 (0.84‐1.88)	.27	1.96 (1.36‐2.84)	<.001
Education				
≤High school graduate	—	—	—	—
Some college	1.81 (1.06‐3.09)	.03	0.86 (0.47‐1.56)	.61
≥College graduate	2.75 (1.56‐4.85)	<.001	1.79 (1.00‐3.20)	.05
Residential area				
Rural (reference)	—	—	—	—
Urban	—	—	1.85 (1.10‐3.13)	.02
Health insurance				
No (reference)	—	—	—	—
Yes	2.40 (1.24‐4.68)	.01	—	—
Caregiving: child				
No (reference)	—	—	—	—
Yes	—	—	1.61 (1.01‐2.57)	.046
Caregiving: spouse				
No (reference)	—	—	—	—
Yes	—	—	2.14 (1.26‐3.65)	.005
Caregiving: cancer				
No (reference)	—	—	—	—
Yes	—	—	1.89 (1.12‐3.19)	.02
Caregiving: chronic conditions				
No (reference)	—	—	—	
Yes	—	—	1.47 (1.03‐2.10)	.04
Access to high-speed internet				
No (reference)	—	—	—	—
Yes	3.04 (1.55‐5.95)	.001	—	—
Having a tablet computer				
No (reference)	—	—	—	—
Yes	1.77 (1.19‐2.62)	.005	1.57 (1.02‐2.42)	.04
Having a smartphone				
No (reference)	—	—	—	—
Yes	2.46 (1.24‐4.89)	.01	2.31 (1.02‐5.22)	.04

a Adjusted for survey cycles, age, and sex; the final sample weight and replicate weights are applied.

b Nagelkerke pseudo-*R*2=0.230.

c OR: odds ratio.

d Nagelkerke pseudo-*R*2=0.200.

e HINTS: Health Information National Trends Survey.

f Not applicable.

#### Health-Related Social Media Use

**Figure 3.**
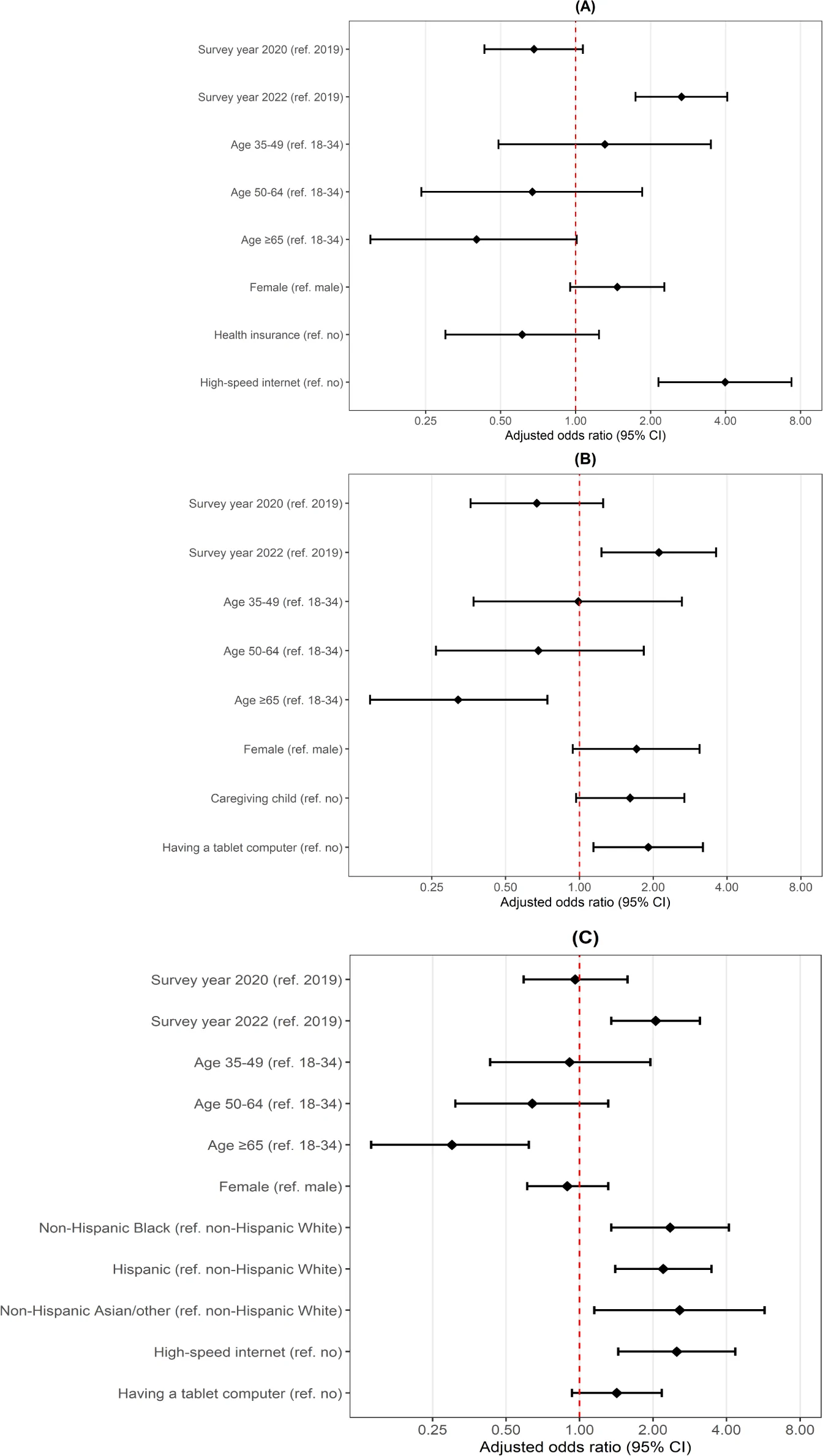
Forest plots of the association between survey cycle/covariates and health-related social media use. (A) Sharing health information, (B) interacting with people, and (C) watching health-related videos.

Compared to HINTS 5 Cycle 3 (2019), caregivers in HINTS 6 (2022) were more likely to share personal or general health information (aOR 2.66, 95% CI 1.74‐4.06; *P*<.001), interact with people who have similar health or medical issues (aOR 2.11, 95% CI 1.23‐3.61; *P*=.007), and watch health-related videos on social media (aOR 2.05, 95% CI 1.35‐3.11; *P*<.001). Caregivers aged 65 years or older had a lower likelihood of interacting with people (aOR 0.32, 95% CI 0.14‐0.74; *P*=.007) and watching health-related videos on social media (aOR 0.30, 95% CI 0.14‐0.62; *P*=.001) compared to younger age groups. Caregivers who were non–Hispanic Black (aOR 2.35, 95% CI 1.35‐4.09; *P*=.003), Hispanic (aOR 2.20, 95% CI 1.40‐3.47; *P*<.001), and non–Hispanic Asian or other (aOR 2.57, 95% CI 1.15‐5.73; *P*=.02) were more likely to watch health-related videos on social media compared to non–Hispanic White. Having access to high-speed internet was associated with a higher likelihood of sharing health information (aOR 3.98, 95% CI 2.15‐7.35; *P*<.001) and watching health-related videos (aOR 2.50, 95% CI 1.44‐4.34; *P*=.001). Having a tablet computer was associated with a higher likelihood of interacting with people (aOR 1.91, 95% CI 1.14‐3.19; *P*=.01) compared to their counterparts. The aORs with 95% CIs and Nagelkerke pseudo-*R*^2^ values of logistic regression models are shown in Table 4, with forest plots displayed in Figure 3.

**Table 4.** Multivariable logistic regression on health-related social media use^a^.

Variables	Sharing health information^b^, OR^c^ (95% CI)	*P* value	Interacting with people^d^, OR (95% CI)	*P* value	Watching health-related videos^e^, OR (95% CI)	*P* value
Survey cycles						
HINTS^f^ 5 Cycle 3 (2019) (reference)	—^g^	—	—	—	—	—
HINTS 5 Cycle 4 (2020)	0.68 (0.43‐1.07)	.09	0.67 (0.36‐1.25)	.21	0.96 (0.59‐1.57)	.86
HINTS 6 (2022)	2.66 (1.74‐4.06)	<.001	2.11 (1.23‐3.61)	.007	2.05 (1.35‐3.11)	<.001
Age group (y)						
18‐34 (reference)	—	—	—	—	—	—
35‐49	1.31 (0.49‐3.49)	.59	0.99 (0.37‐2.62)	.98	0.91 (0.43‐1.95)	.81
50‐64	0.67 (0.24‐1.85)	.44	0.68 (0.26‐1.83)	.45	0.64 (0.31‐1.31)	.22
≥65	0.40 (0.15‐1.01)	.05	0.32 (0.14‐0.74)	.007	0.30 (0.14‐0.62)	.001
Sex						
Male (reference)	—	—	—	—	—	—
Female	1.47 (0.95‐2.27)	.08	1.71 (0.94‐3.09)	.08	0.89 (0.61‐1.31)	.56
Race/ethnicity						
Non–Hispanic White (reference)	—	—	—	—	—	—
Non–Hispanic Black	—	—	—	—	2.35 (1.35‐4.09)	.003
Hispanic	—	—	—	—	2.20 (1.40‐3.47)	<.001
Non–Hispanic Asian or other	—	—	—	—	2.57 (1.15‐5.73)	.02
Health insurance						
No (ref.)	—	—	—	—	—	—
Yes	0.61 (0.30‐1.24)	.17	—	—	—	—
Caregiving: child						
No (reference)	—	—	—	—	—	—
Yes	—	—	1.61 (0.97‐2.68)	.07	—	—
Access to high-speed internet						
No (reference)	—	—	—	—	—	—
Yes	3.98 (2.15‐7.35)	<.001	—	—	2.50 (1.44‐4.34)	.001
Having a tablet computer						
No (reference)	—	—	—	—	—	—
Yes	—	—	1.91 (1.14‐3.19)	.01	1.42 (0.93‐2.17)	.10

a Adjusted for survey cycles, age, and sex; final sample weight and replicate weights are applied.

b Nagelkerke pseudo-*R*2=0.165.

c OR: odds ratio.

d Nagelkerke pseudo-*R*2=0.128.

e Nagelkerke pseudo-*R*2=0.166.

f HINTS: Health Information National Trends Survey.

g Not applicable.

## Discussion

### Principal Findings

This study examined trends in digital health engagement behaviors—specifically accessing online medical records and using social media for health-related purposes—among unpaid family caregivers in the United States using 3 cycles of nationally representative HINTS data collected before, during, and after the COVID-19 pandemic. Overall, caregivers’ engagement with digital health technologies increased substantially following the pandemic, particularly with respect to accessing online medical records for both themselves and their care recipients.

Consistent with trends observed in the general US population [30], in this study, family caregivers surveyed in the postpandemic cycle were significantly more likely to access online medical records for both themselves and their care recipients than those surveyed prior to the pandemic. This acceleration has been attributed to the rapid expansion of digital health technologies during the pandemic and to regulatory efforts—most notably the 21st Century Cures Act—that require health care organizations and health IT developers to provide patients with easier electronic access to their health information [31]. In addition, this finding is consistent with prior work showing that caregiving became a stronger predictor of online medical record use after COVID-19 than before the pandemic [32]. As caregiving responsibilities intensified during the pandemic—amid disruptions in in-person care and increased reliance on remote communication—caregivers may have increasingly relied on electronic health records to manage appointments, medications, test results, and care coordination. An important key contribution of this study is its longitudinal comparison of caregivers’ access to both their own and their care recipients’ online medical records across survey cycles spanning the pandemic. Prior studies have often focused on patient portal use among patients themselves or relied on cross-sectional designs [33-35]. By explicitly examining caregivers’ access to care recipients’ records, this study addresses a significant gap in the evidence, as caregivers frequently rely on online medical records to manage medications, monitor test results, and communicate with providers on behalf of care recipients [36-38].

In addition to survey cycles, several demographic and socioeconomic factors were associated with disparities in online medical record access among family caregivers. Higher educational attainment and health insurance coverage were associated with greater access to caregivers’ own records, consistent with previous studies among US adults [33,34,39]. These disparities may reflect differences in digital and health literacy, frequency of health system interactions, and familiarity with online patient portals [40,41]. Caregivers residing in urban areas were more likely to access care recipients’ online medical records than those living in rural areas, a pattern that mirrors broader rural-urban gaps in digital health engagement and infrastructure [35,42,43].

The caregiving context also shaped digital health engagement. Caring for a spouse or child, as well as for individuals with cancer or chronic conditions, was associated with a greater likelihood of accessing care recipients’ online medical records, but not caregivers’ own records. These findings suggest that caregivers’ use of online medical records is driven primarily by caregiving responsibilities and clinical complexity rather than personal health needs. Similar patterns have been observed in studies of caregivers managing serious or chronic illnesses, where electronic access to health information supports coordination, monitoring, and decision-making [36,44].

Access to high-speed internet and ownership of mobile devices were strong facilitators of online medical record use among caregivers in this study. These findings align with broader evidence that broadband access and mobile device availability are foundational to equitable digital health engagement [30,35,43,45]. Mobile-enabled access may be particularly important for caregivers, who often manage care across multiple settings and time constraints. Prior studies suggest that individuals who use mobile devices or health apps access health information more frequently and consistently than those who rely solely on websites [46]. Our results highlight the need for policies that focus on increasing high-speed internet access. Enhancing this digital infrastructure is increasingly critical in areas where digital health care needs and remote access are particularly important. In contrast to the steady increase in online medical record access, caregivers’ health-related use of social media followed a nonlinear pattern, declining during the early pandemic period and increasing substantially in the postpandemic cycle. Following the pandemic, caregivers were more likely to share health information, interact with others with similar health concerns, and watch health-related videos on social media. Social media platforms have become increasingly important sources of health information and peer support, particularly during and after the COVID-19 pandemic [47,48].

Disparities and cultural factors were also evident in health-related social media use. Older caregivers (aged ≥65 y) were less likely to interact with others or watch health-related videos on social media compared with younger caregivers (aged 18‐34 y). Access to high-speed internet and ownership of mobile devices were associated with greater engagement. These differences may influence the reach and effectiveness of social media–based health interventions, highlighting the need for tailored strategies that consider age and digital access [47,49]. In addition, non–Hispanic White caregivers were less likely to engage in certain health-related social media activities than non–Hispanic Black, Hispanic, and non–Hispanic Asian or other caregiver groups. National survey data show that racial and ethnic minority adults demonstrate equal or higher engagement with social media platforms compared with White adults [50]. Prior research also indicates that Black and Hispanic caregivers often rely more heavily on family-centered and kin-based support networks compared with non–Hispanic White caregivers [51,52]. Together, these patterns suggest that cultural differences are factors to consider, and social media may function as an extension of kin- and community-based information exchange among racial and ethnic minority caregivers. At the same time, the growing reliance on social media for health information raises concerns about health misinformation, underscoring the importance of digital health literacy and the promotion of credible information sources [53].

Notably, existing research on social media use among family caregivers has largely emphasized qualitative analyses of online interactions or associations with caregiver outcomes. Population-level studies examining trends and correlations of health-related social media use among caregivers remain limited. This study contributes to the literature by documenting temporal trends and identifying key sociodemographic and technological factors associated with caregivers’ health-related social media engagement.

### Limitations

This study has several limitations. First, the datasets lacked detailed information on caregiving intensity, duration, and caregiving tasks, which may influence digital health engagement. Second, harmonizing variables across survey cycles requires recategorization, which may have introduced measurement inconsistencies. Third, the repeated cross-sectional design does not allow the assessment of longitudinal changes within the same individuals. Finally, the analysis was limited to selected digital health engagement behaviors and did not capture the full range of caregivers’ digital health activities.

### Conclusions

There was a clear increase in US family caregivers’ access to online medical records and health-related use of social media, following the COVID-19 pandemic. However, disparities by age, race/ethnicity, geography, education, insurance coverage, and digital access persist. These findings highlight the need for caregiver-centered, equity-focused digital health strategies and suggest that future technology-based interventions should account for the diverse characteristics and needs of family caregivers. The findings provide insight into the importance of digital health engagement among caregiver populations, with consideration of equity and cultural factors at the policy level, particularly for caregivers living in rural areas and/or those with limited access to broadband internet and mobile connectivity, which may affect digital health access. Future research should also examine caregivers’ access to and use of emerging digital tools such as AI-based conversational agents (eg, GPT-powered chat tools) to understand their potential roles in health information seeking, decision support, and emotional support, as well as the risk of misinformation associated with digital platforms, including algorithm-driven short-form video apps (eg, TikTok).

## Supplementary material

10.2196/94390 Checklist 1 STROBE checklist.

## Abbreviations

aOR: adjusted odds ratio
HINTS: Health Information National Trends Survey
IRB: Institutional Review Board
OR: odds ratio
STROBE: Strengthening the Reporting of Observational Studies in Epidemiology
